# Xanthogranulomatous pyelonephritis combined with emphysematous pyelonephritis: A rare case report

**DOI:** 10.22088/cjim.12.0.505

**Published:** 2021

**Authors:** Emadoddin Moudi, Mohammadmehdi Darzi, Sepehr Ramzani, Abazar Akbarzadeh Pasha

**Affiliations:** 1 Department of Urology, Babol University of Medical Sciences, Babol, Iran; 2 Cancer Research Center, Health Research Institute, Babol University of Medical Sciences, Babol, Iran; 3 Clinical Research Development Center, Shahid Beheshti Hospital, Babol University of Medical Sciences, Babol, I.R.Iran; 4 Student Research Committee, Babol University of Medical Sciences, Babol, Iran; 5 Faculty of Medicine, Babol University of Medical Science, Babol, Iran

**Keywords:** Xanthogranulomatous pyelonephritis, Emphysematous pyelonephritis, Staghorn calculi

## Abstract

**Background:**

Xanthogranulomatous pyelonephritis (XGP) is a rare and intense type of chronic kidney infection characterized by subversion of the renal tissue and its replacement by lipid- laden macrophages. XGP combined with emphysematous pyelonephritis(EPN) is rare and up until now only 7 cases with these presentations have been reported; so abundant clinical skills and appropriate radiographic imaging is required to reach the correct diagnosis. In this report, we present a case with two uncommon variants of pyelonephritis.

**Case Presentation:**

A 55-year-old female presented with a history of type 2 diabetes mellitus, and a stroke leading to a left-sided hemiplegia state for 7 years, coronary artery bypass grafting(CABG), hypertension(HTN) , seizure, progressive fatigue, loss of appetite , fecal and urinary incontinence and right costovertebral angle tenderness. According to clinical signs, symptoms and documentation of gas within the renal parenchyma on computed tomography (CT) of abdomen, of EPN diagnosis was suggested; however histopathologic evaluation showed acute emphysematous pyelonephritis on chronic xanthogranulomatous pyelonephritis.

**Conclusion:**

EPN can emerge in a patient suffering from XPG which would add to the severity of the situation. In the case presented, concurrent underlying diseases such as diabetes mellitus, stroke, CABG, HTN along with severe fatigue and loss of appetite existed. Surgical treatment produces dramatic results.

XGP is an infrequent inflammatory process of renal parenchyma and circumambient tissues which occur in the presence of chronic obstruction and indicated pathologically by the appearance of xanthoma cells (1). The kidney affected is usually associated with stones and hydronephrosis (2), present in middle-aged women and with an occurrence rate of sub 1% of chronic pyelonephritis patients. The characteristic symptoms of patients with XGP are flank pain and fever (3, 4). XGP can be found in 2 classifications: the focal form and diffuse form. The diffuse form of XGP manifests extrarenal complications (5, 6). If not treated immediately, the diffuse form of XGP can lead to a lethal state, containing perinephric inflammation, psoas abscess, nephrocutaneous fistula, and renocolic fistula (7–9). Antibiotic therapy in XGP patients does not affect pathological process , thus total nephrectomy should be considered (10). 

In our paper, we present a 55-year old female suffering from progressive fatigue incontinence and costovertebral angle tenderness, who was found to have right-sided emphysematous pyelonephritis. This finding was confirmed by CT, eventually she underwent a right-sided total nephrectomy and histological analysis reported acute emphysematous pyelonephritis on chronic xanthogranulomatous pyelonephritis with abscess formation.

According to the ethical code of IR.MUBABOL.REC.1399.448 we report a rare case about combined occurrence of XGP with EPN.

## Case presentation

In April 2020, a bedridden 55-year-old woman from Babol, Mazandaran with a medical background of type 2 diabetes mellitus, and a stroke leading to a left-sided hemiplegia state for 7 years, CABG, HTN and seizure presented to our emergency department. She was admitted with progressive fatigue, loss of appetite and fecal and urinary incontinence temperature of 36°C and pulse of 90 bpm. Physical examination findings were bilateral lower abdomen and costovertebral angle tenderness on the right side.Laboratory findings showed initial WBC count of 6.6×103/µL, hemoglobin of 7 g/dL with microcytic anemia, creatinine of 1.3 mg/dL and urinalysis as indicative of urine infection.

**Fig 1:**
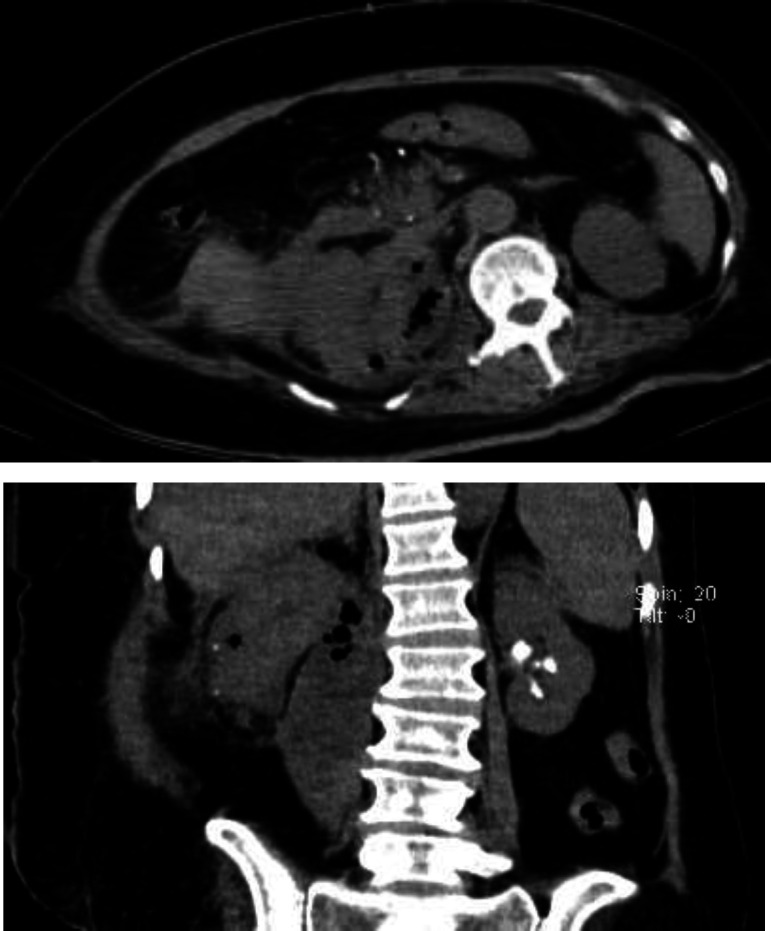
Abdominopelvic CT scan

Computed tomography revealed a left kidney with a staghorn stone, right kidney with two small calculi thickening of the pelvic wall.Two small calculi were identified within the collecting system and also the right calyx in addition to enlarged destroyed renal parenchyma with small bubbles of gas and fluid collections. Also, there were multiple low-density areas throughout the kidney, suggestive of necrosis or abscess, penetrating the psoas muscle (fig 1).

**Fig 2:**
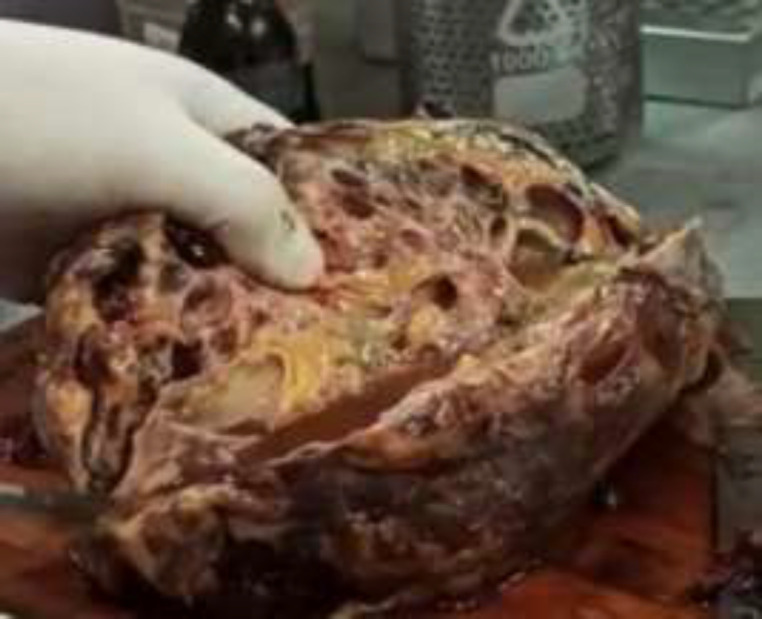
Nephrectomy specimen after longitudinal incision

The antibiotic regimen of choice was intravenous ceftriaxone and gentamicin, due to urine culture that was cephalosporins sensitive E. coli and Klebsiella oxytoca (KO). Eventually, she underwent a right-sided total nephrectomy 5 days after initiation of antibiotic therapy and ureteral stenting due to the worsening of the symptoms (fig 2). Pathological examination reported acute EPN on chronic XGP with abscess formation. 

The patient had a significant clinical improvement and was discharged 5 days after surgery prescribed with oral antibiotics.

## Discussion

XGP was first reported by Schlagenhaufer in 1916 (11). XGP is an unusual type of unilateral chronic kidney infection presenting with renal mass, accompanied by stones or urinary tract obstruction (12). The types of focal, segmental and diffuse XGP have been reported(13). The diffuse form can be restricted to the kidney or expanded to the renal pelvic, fat inside of Gerota fascia, or retroperitoneal organs (14). Common symptoms of XGP include costovertebral angle tenderness, urinary frequency, dysuria, nocturia, and burning (14). In a study, most symptoms and signs were abdominal and flank pain dysuria, urinary frequency, nocturia, palpable mass, increase in blood WBC and fever (15). Computed tomography can be the best imaging study to identify XGP (16), but XGP has unusual finding on computed tomography so diagnosis or differential diagnosis of XGP is usually effortful for the physician (17). Common findings of diffuse form of XGP in computed tomography are as follows: Collecting system dilation, kidney stones, pyonephrosis, collection in renal tissue and perirenal abscess. Focal type could be reported as a pseudotumoral lesion (17). Lipid laden foamy macrophage collection can be recognized by MRI on T1 weighed images as high intensity signal (18). DTPA renal scan and intravenous urography have low sensitivity for XGP diagnosis (19).

Emphysematous pyelonephritis (EPN) is a severe necrotizing infection of the renal parenchyma (20). Such infections can arise because of E.coli and klepsiella pneumoniae activity on the basis of diabetes and/or obstruction, and produce gas by acid fermentation of glucose(21). EPN occasionally occurs in patients with urinary tract obstruction (22). By producing gas within and around the kidney, EPN can prove to be a lethal infection (11). EPN severity has been classified by Hoang(23). With the increase in numbers of ultrasonography and CT scan, more cases of EPN are being diagnosed (24).

In this case, not unlike most of the previous ones, the disease emerged in a female diabetic patient. She showed noticeable levels of fatigue. EPN was diagnosed before surgery and XGP after the pathology results came in; as in SV Punekar’s report (25). In our case report as in most cases, more than one micro-organism was identified.

In some reports, the disease was accompanied by fistula extension to the neighboring organs, muscle and flank areas (26). In our case, the psoas muscle was involved and unlike other cases, medical management and ureteral stenting did not lead to a better situation, but surgical extirpation resulted in dramatic recovery.
